# [Retracted] Role of the EZH2/miR-200 axis in STAT3-mediated OSCC invasion

**DOI:** 10.3892/ijo.2025.5740

**Published:** 2025-03-17

**Authors:** Yu Wang, Wenyu Guo, Zhaoqing Li, Yansheng Wu, Chao Jing, Yu Ren, Minghui Zhao, Lingping Kong, Chao Zhang, Jiabin Dong, Yu Shuang, Shanshan Sun, Jinliang Chen, Chuanqiang Wu, Yu Qiao, Xin Qu, Xudong Wang, Lun Zhang, Rui Jin, Xuan Zhou

Int J Oncol 52: 1149-1164, 2018; DOI: 10.3892/ijo.2018.4293

Following the publication of the above paper, and a corrigendum that has already been published (doi.org/10.3892/ijo.2023.5528) to attend to errors that were made in the assembly of Figs. 2 and 8, it was drawn to the Editor's attention by a concerned reader that two pairs of data panels featuring overlapping data existed comparing Figs. 1C and 2D in the above paper with Fig. 2 in a paper published in the journal *Molecular Cancer Therapeutics*, which was published before the above paper had been submitted, even though this earlier paper did contain many of the same authors.

Given that this further issue has come to light, and owing to the number of duplications of data and errors made in assembling figures that have now been identified in this paper, the Editor of *International Journal of Oncology* has decided that it should be retracted from the Journal on account of a lack of confidence in the originally presented data. The authors were asked for an explanation to account for these concerns, but the Editorial Office did not receive a reply. The Editor apologizes to the readership for any inconvenience caused.

